# Age-based dynamic changes of phylogenetic composition and interaction networks of health pig gut microbiome feeding in a uniformed condition

**DOI:** 10.1186/s12917-019-1918-5

**Published:** 2019-05-24

**Authors:** Shanlin Ke, Shaoming Fang, Maozhang He, Xiaochang Huang, Hui Yang, Bin Yang, Congying Chen, Lusheng Huang

**Affiliations:** State Key Laboratory of Pig Genetic Improvement and Production Technology, Jiangxi Agricultural University, 330045 Nanchang, People’s Republic of China

**Keywords:** Co-occurrence network, Enterotype, Age, Gut microbiota, 16S rRNA, Pig

## Abstract

**Background:**

The gut microbiota impacts on a range of host biological processes, and the imbalances in its composition are associated with pathology. Though the understanding of contribution of the many factors, e.g. gender, diet and age, in the development of gut microbiota has been well established, the dynamic changes of the phylogenetic composition and the interaction networks along with the age remain unclear in pigs.

**Results:**

Here we applied 16S ribosomal RNA gene sequencing, enterotype-like clustering (Classification of the gut microbiome into distinct types) and phylogenetic co-occurrence network to explore the dynamic changes of pig gut microbiome following the ages with a successive investigation at four ages in a cohort of 953 pigs. We found that Firmicutes and Bacteroidetes are two predominant phyla throughout the experimental period. The richness of gut microbiota was significantly increased from 25 to 240 days of age. Principal coordinates analysis showed a clear difference in the gut microbial community compositions between pre-weaning piglets and the pigs at the other three age groups. The gut microbiota of pre-weaning piglets was clearly classified into two enterotypes, which were dominated by *Fusobacterium* and *p-75-a5*, respectively. However, *Prevotella* and *Treponema* were the main drivers of the enterotypes for pigs at the age of 80, 120 and 240 days. Besides the piglets, even some adult pigs switched putative enterotypes between ages. We confirmed that the topological features of phylogenetic co-occurrence networks, including scale, stability and complexity were increased along with the age. The biological significance for modules in the network of piglets were mainly associated with the utilization of simple carbohydrate and lactose, whereas the sub-networks identified at the ages of 80, 120 and 240 days may be involved in the digestion of complex dietary polysaccharide. The modules related to the metabolism of protein and amino acids could be identified in the networks at 120 and 240 days. This dynamic change of the functional capacities of gut microbiome was further supported by functional prediction analysis.

**Conclusions:**

The present study provided meaningful biological insights into the age-based dynamic shifts of ecological community of porcine gut microbiota.

**Electronic supplementary material:**

The online version of this article (10.1186/s12917-019-1918-5) contains supplementary material, which is available to authorized users.

## Background

The microbial community of the mammalian gastrointestinal tract harbors a complex and dynamic ecosystem, which is populated with as many as trillions of microbes, including viruses, bacteria, archaea, fungi and protists [1]. Gut microbiota executes numerous vital functions for homeostasis ranging from harvesting essential nutrients, regulating host metabolism and maintaining the immune system [2]. The establishment and maintenance of the gut microbial ecosystem are crucial to host health because they ensure the persistent occurrence of functions associated with those beneficial microbes [3]. The gut microbiota can be influenced by diet, age, host genetics, and many environmental factors [4–7]. A previous study indicated clear differences in the composition of gut microbiota among infants, toddlers, adults and the elderly [8]. The gut microbiota of newborns undergoes substantial modulation, in which facultative and strict anaerobes gradually dominate the community by replacing aerobes [9]. The age-dependent shifts in gut microbiome reflect the change from the primary lactate metabolism to the enhancement of plant polysaccharide metabolism ability [10]. The bacterial diversity of human gut microbiota increases with age and the phylogenetic composition evolves towards an adult-like configuration within the first three years after birth [4]. The most noticeable feature in the gut microbial composition between young and elderly individuals is an alteration in the ratio of Firmicutes to Bacteroidetes, in which the young adults have a higher proportion of Firmicutes and the older adults show a higher proportion of Bacteroidetes [11]. In pigs, previous reports indicated that the alpha-diversity and the taxonomic composition of intestinal microbiota were significantly altered with the growth of pigs [12–15]. A study in commercially raised pigs found a significant association between aging and an increasing measure of richness and diversity as well as distinct changes of the core microbiota [16].

The complexity of intestinal flora is reflected in both of community structure and function capacity due to their dynamic nature and compositional variability. Microbes in gastrointestinal tract form a complex ecological system with various symbiotic relationships, rather than a simplified collection of independent individuals [17]. The interactions between or among organisms in a microecological system can be characterized into the relationships of mutualism, commensalism, amensalism, predation and competition [18]. The co-occurrence patterns of microorganisms and the microbial relationships are essential for community assembly and stability [19], and can deduce the various effects on the host health. The application of system and network theory can facilitate the analyze of the composition and interaction of gut microbiota, enhancing our understanding of its complex ecological characteristics [20]. Although the classical longitudinal analysis captures the changes of gut microbial composition in outcomes over time, the information about dynamic changes of the interaction networks between bacteria remains unknown. To our knowledge, there has been no systematic study about longitudinal dynamics of the phylogenetic composition and the interaction network of gut microbiota in pigs, especially, in an experimental pig cohort collected fecal samples at multiple ages. Furthermore, due to the similarity of the pig and human in gastrointestinal system and metabolism physiology [21], swine has been considered as one of the ideal models for studying the dynamic natures of gut microbiome.

In this study, we employed enterotype-like clustering analysis and co-occurrence network analysis to investigate the age-based dynamic shifts of pig gut microbiota in an experimental pig population collected the fecal samples at four different ages. Our results provided biological insights into the organization, function and evolution of pig gut microbial community following the age.

## Methods

### Experimental animals

The experimental cohort was sourced from one pig farm of Jiangxi Agricultural University in Nanchang, Jiangxi Province and comprised of 953 pigs from a F_6_ population of heterogeneous pig cross, which had been constructed with eight founder breeds, including four Western commercial pig breeds of Landrace, Large White, Duroc and Pietran, and four Chinese indigenous pig breeds of Erhualian, Bamaxiang, Tibetan and Laiwu. All pigs were raised at the experimental pig farm of Jiangxi Agricultural University in Nanchang, Jiangxi. Piglets were housed with their mothers during suckling period and creep feed was provided during the last week of lactation (Additional file 1: Table S1). After the weaning at 28 days, all experimental pigs were transferred to a uniform fattening house and fed two times a day with formula diets (Additional file 1: Table S1). Water was available ad libitum from nipple drinkers. All boars were castrated at 80 days. All experimental animals used in this study were healthy and did not receive any antibiotic treatments within two months before fecal sample collection. The experimental pigs were slaughtered at a commercial slaughterhouse at 240 days of age by bleeding after electrical stunning.

### Collection of fecal samples and DNA extraction

Fecal samples were collected at the ages of 25, 120 and 240 days, which represented the time of preweaning, mid-stage of fattening and slaughtering. A total of 1417 fecal samples were harvested from the rectum of experimental pigs in the cohort, including 175 samples collected at the age of 25 days (from 92 females and 83 entire males), 551 samples at the age of 120 days (from 302 females and 249 castrated males) and 691 samples at the age of 240 days (from 377 females and 314 castrated males). In another study, to investigate the effect of castration on the phylogenetic composition of gut microbiota, we collected additional 64 fecal samples (from 36 females and 28 entire males) in the same experimental cohort at the age of 80 days (boars were castrated immediately after collected the fecal samples). To analyze dynamic changes of gut microbiota at more age times, the bacterial composition data of these 64 samples were also included in this study. The detailed information about sampling is shown in the Additional file 2: Figure S1.

All samples were collected in sterile plastic centrifuge tubes and deep frozen in liquid nitrogen. After transported to the laboratory, the samples were stored at − 80 °C freezer until use. DNA extraction from the fecal samples was performed using QIAamp DNA Stool Mini Kit (Qiagen, Germany) following manufacturer’s instructions (McOrist et al., 2002). The quality and integrity of the DNA samples were checked by electrophoresis with 0.8% agarose gel, and DNA concentrations were measured with a ND-1000 spectrophotometer (Nanodrop Technologies, USA).

### Amplification and sequencing of bacterial 16S rRNA gene

The V3-V4 region of 16S rRNA gene was amplified by PCR with the barcode fusion forward primer 338F [ACTCCTACGGGAGGCAGCAG] and the reverse primer 806R [GGACTACHVGGGTWTCTAAT] under the melting temperature of 55 °C with 28 cycles. For the bacterial composition data of 64 samples harvested at the age of 80 days mentioned above, only V4 hypervariable region of 16S rRNA gene was amplified by the fusion primers 515F [GTGCCAGCMGCCGCGGTAA] and 806R [GGACTACHVGGGTWTCTAAT] under the melting temperature of 56 °C with 30 cycles. The sequencing of the PCR amplicons was performed with a 250-bp paired-end procedure on an Illumina MiSeq platform (Illumina, USA) according to the manufacturer’s manuals.

### Bioinformatics processing of sequence reads

To obtain the clean sequence reads, the primer and barcode sequences, and the low-quality reads were excluded from further analysis. FLASH (version 1.2.11) was used to assemble the paired-end clean reads into tags [22]. To avoid the bias generated by the different sequencing depth [23], we rarefied the library size to 10,000 tags per sample. Unique bacterial sequences with 97% of the sequence similarity were clustered into operational taxonomic unit (OTU) using the QIIME software (the toolbox for Quantitative Insights Into Microbial Ecology) [24], which uses UCLUST (an algorithm to cluster sequence reads based on similarity) to perform the clustering [25]. OTUs were matched to bacteria by using a primer-specific version of the GreenGenes reference database (version 13.5) [26]. We filtered out those OTUs which had relative abundance less than 0.01% and were presented in less than 1% of the experimental pigs from further analyses. The alpha-diversity indexes of chao1, ACE, observed species, Simpson and Shannon were calculated by Mothur software (Version 1.42.0) [27]. The comparison of the relative abundances of bacterial taxa and the alpha-diversity indexes between two age groups was performed by Wilcoxon *t*-test. The *P* values were adjusted the multiple tests by Benjamini-Hochberg (BH) method with the threshold of false discovery rate (FDR) ≤ 5%. In order to evaluate the effects of host age and sex on microbial composition of fecal samples, a Bray-Curtis similarity matrix was constructed based on the relative abundances of OTUs. We split the sequences of V3-V4 region of 16S rRNA gene into the sequences containing only V4 region for the samples at 25, 120 and 240 days of age. And then, all sequence data of the V4 region were incorporated together for further calculation of Bray-Curtis matrix. Principal coordinated analysis (PCoA) of Bray-Curtis distances was performed using the R function pcoa.

### Enterotype-like clustering

The enterotype-like clustering analysis of fecal microbiota at four ages was separately performed with the method described previously [28]. Briefly, we calculated Jensen-Shannon divergence (JSD) distance for the relative abundances of bacterial taxa at the genus level by applying the Partitioning Around Medoids (PAM) method. The optimal number of clusters and the groups’ robustness were evaluated with CalinskiHarabasz (CH) index and silhouette value, respectively. To identify the genera with different abundances between the two enterotypes, a linear discriminant analysis (LDA) effect size (LEfSe) analysis was performed under the condition ɑ = 0.01, with an LDA score of at least 3 [29]. Sparse Correlations for Compositional data (SparCC) was employed to determine co-abundance (positive) and co-exclusion (negative) relationships between genera based on their relative abundances [30]. Significant correlations between bacterial genera were detected using the partial correlation and information theory (PCIT) algorithm [31]. The main absolute correlations (top 15%) were transformed into links between two genera in the genus network and the networks were visualized in Cytoscape (version 3.4.0).

### Construction of phylogenetic interaction networks

We filtered out those OTUs which had less than 0.05% of relative abundance and were presented in less than 10% of the tested samples from further interaction network analysis. Phylogenetic co-occurrence networks of porcine fecal microbiota at the ages of 25, 80, 120 and 240 days were separately inferred based on the SparCC algorithm [30]. Significant correlations due to the compositional structures of OTUs were detected using the PCIT algorithm [31]. The confidence of the interactions between nodes was established with > 0.55 of absolute sparse correlation coefficient. We used Cytoscape (version 3.4.0) to visualize co-occurrence networks and calculate their topological characteristics including densities, clustering coefficients and scale-free properties [32]. The clustering of sub-modules was based on the vertex weighting by local neighborhood density and outward traversal from a locally dense seed node according to given parameters. Assessment of biologically important modules in OTU networks was performed using Molecular Complex Detection (MCODE) plugin in the Cytoscape software [33]. To further compare the topological characteristics of main modules in the interaction networks, we used Network Analysis Profler (NAP) to numerically calculate the topological features and metrics of networks (e.g. average eccentricity, average number of neighbors, centralization betweenness, centralization closeness and centralization degree) [34].

### Prediction and comparison of functional capacities of fecal microbiome among four age groups

To compare the potential function capacities of fecal microbiome among four age groups, we used the phylogenetic investigation of communities by reconstruction of unobserved states (PICRUST) algorithm to obtain the function profiles of bacterial community [35]. Functional genes were categorized into KEGG pathways. The relative abundance of each subclass term of the KEGG pathways was calculated by summing the abundances of functional genes that were annotated to the functional subsystem. Tukey-Kramer post-hoc test was used for pairwise comparisons of potential function capacities of fecal microbiomes among four age groups. Story’s FDR was used to correct the multiple tests. All tests were performed using Statistical Analysis of Metagenomic Profiles (STAMP) software [36].

## Results

### Longitudinal dynamics of fecal microbial composition and richness

**Fig. 1:**
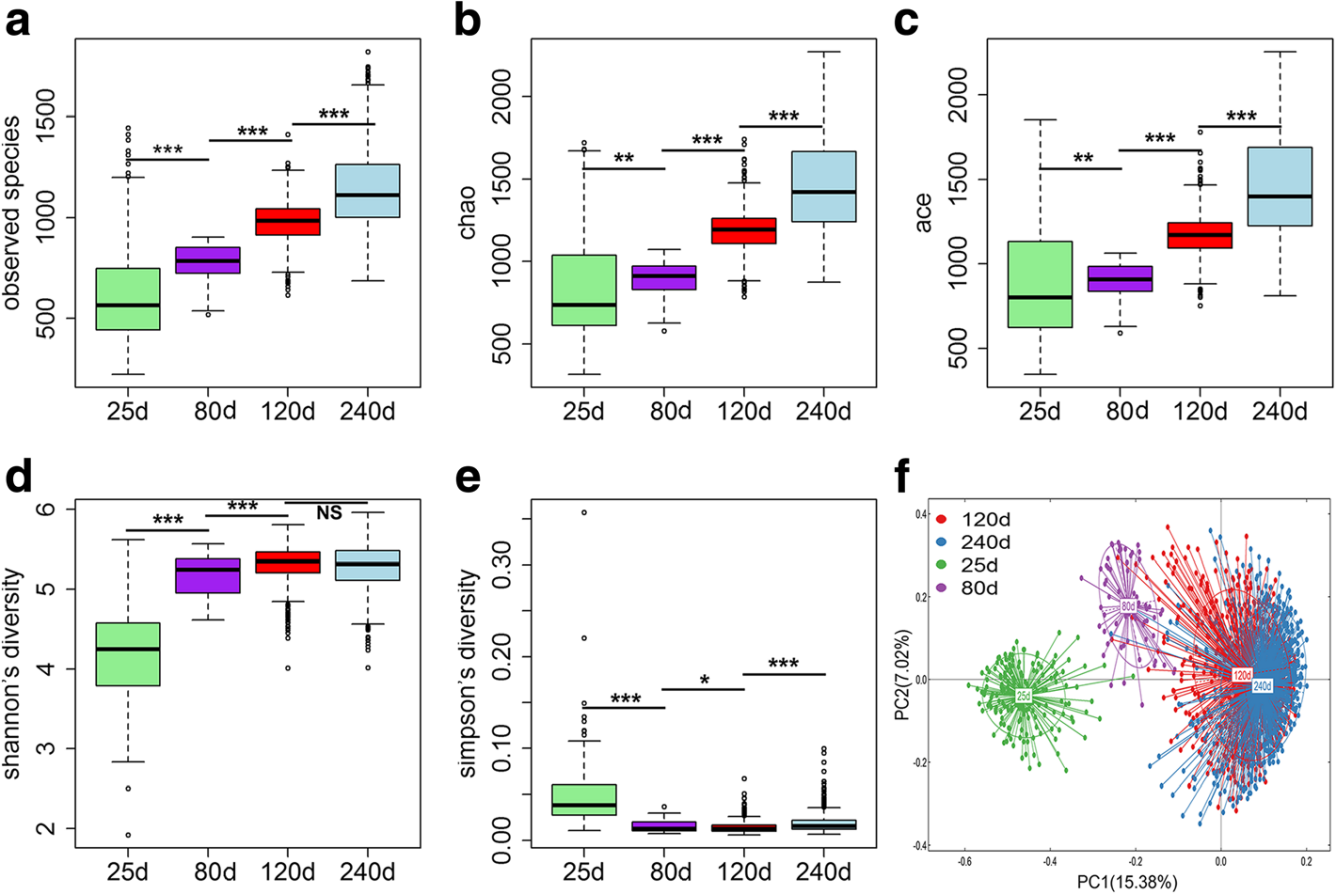
Comparison of the diversity of gut microbiome among four ages. The gut microbial richness was estimated by observed species, chao and ace index. The diversity was evaluated by Shannon and Simpson index. PCoA of the fecal bacterial communities was based on the Bray-Curtis similarity matrix. (**a**) Observed species. (**b**) Chao. (**c**) ACE. (**d**) Shannon index. (**e**) Simpson index. (**f**) PCoA plot based on the Bray-Curtis similarity matrix. Wilcoxon *t*-test was used to compare the richness and diversity of gut microbiota between two successive ages (NS: not significant, *P ≤ 0.05, **P ≤ 0.01, ***P ≤ 0.001)

The total numbers of high-quality sequence tags for samples at the ages of 25, 80, 120 and 240 days were 5,746,871 (an average of 32,839 tags per sample), 1,082,852 (an average of 16,919 tags per sample), 16,825,154 (an average of 30,536 tags per sample) and 19,749,079 (an average of 28,580 tags per sample), respectively. A total of 3392, 2344, 3401 and 3609 OTUs were obtained. To investigate the dynamic shifts of fecal microbiota structure along with the age, the alpha-diversity of fecal microbiota was compared among four ages using the Chao1, ACE, observed species, Shannon and Simpson index. Interestingly, the experimental pigs showed the continuously increased indexes of Chao1, ACE and observed species following the age (Fig. 1A-C). Shannon index, which reflects the richness and evenness of bacterial taxa, was increased markedly from the age of 25 to 120 days, but showed no significant difference between the ages of 120 and 240 days (Fig. 1D). The Simpson index was significantly decreased from 25 to 120 days, but slightly increased from 120 to 240 days (Fig. 1E). This result suggested that the richness of gut microbiota was continuously increased from 25 to 240 days, but the highest diversity was achieved at 120 days. PCoA analysis based on the relative abundances of OTUs revealed the distinctly microbial compositions between pre-weaning piglets and the pigs at the other three ages (Fig. 1F). The bacterial composition of fecal samples at the age of 80 days was more similar to that at 120 and 240 days. However, no distinct difference was observed in fecal bacterial structures between the ages of 120 and 240 days.

**Fig. 2:**
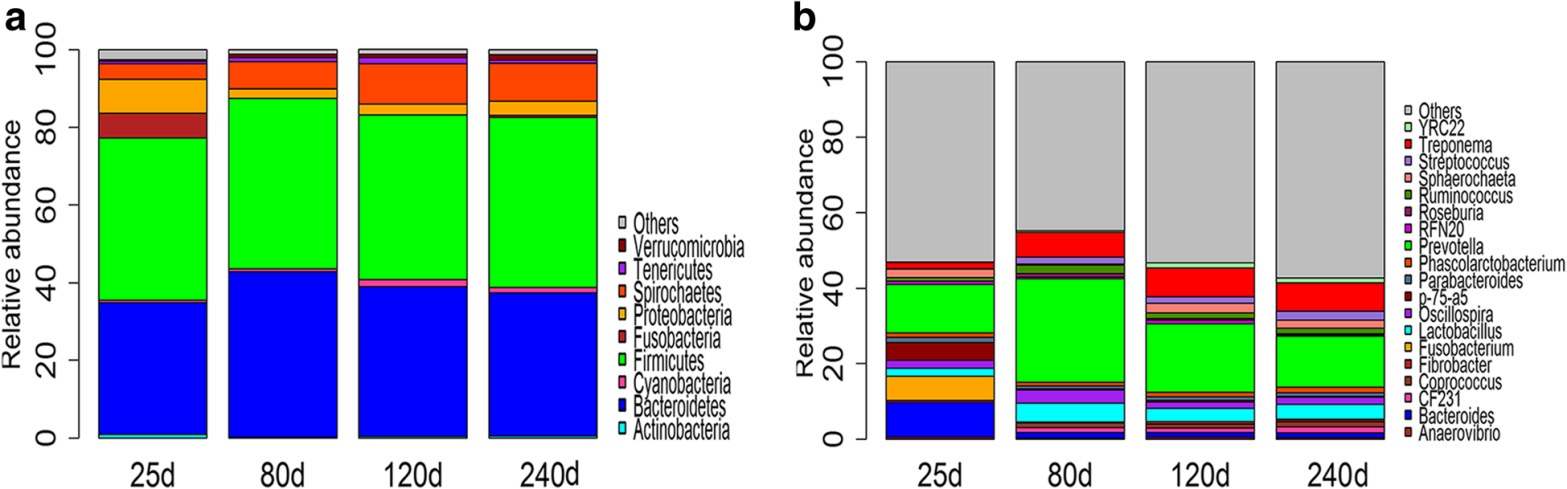
Histograms of the relative abundances of gut microbial taxa at four ages. (**a**) At the phylum level. (**b**) At the genus level. The X-axis shows the ages of the experimental pigs, and the Y-axis represents the relative abundances of bacteria

We further evaluated the age-based dynamic changes of microbial composition at the taxonomic level. The phylogenetic compositions of fecal microbiota at the phylum level at four ages are shown in Fig. 2A. Firmicutes was the most predominant phylum at all four age stages, but had slightly less abundance in piglets (25 days) compared with that in pigs at the other three ages (*P* > 0.05). The relative abundance of Bacteroidetes was increased from the age of 25 to 80 days (*P* < 0.0001), but subsequently decreased after 80 days (*P* < 0.01). The relative abundances of Proteobacteria and Fusobacteria were decreased dramatically after the age of 25 days (*P* < 0.0001). At the genus level, the fecal microbiota of piglets was dominated by *Bacteroides*, *Fusobacterium*, *p-75-a5* and *Prevotella*, whereas *Lactobacillus*, *Prevotella*, *Ruminococcus* and *Treponema* were the core bacteria for fecal samples at 80, 120 and 240 days of age (Fig. 2B). The relative abundances of three predominant genera in pre-weaning pigs, including *Bacteroides*, *Fusobacterium* and *p-75-a5* were significantly decreased after 25 days of age (*P* < 0.0001; Fig. 2B). The relative abundances of *Lactobacillus*, *Prevotella* and *Ruminococcus* were significantly increased from 25 to 80 days of age (*P* < 0.0001), but subsequently decreased from the age of 80 to 120 days (*P* < 0.05). However, there was no significant difference between 120 and 240 days. The proportion of *Treponema* was significantly increased from 25 to 120 days (*P* < 0.05), but showed no significant difference between 120 and 240 days (*P* > 0.05).

Besides age, we also evaluated the effect of host sex on gut microbial community structures at all four age stages. Although the samples harvested at 80 days of age showed a slight difference in bacterial compositions between gilts and entire boars, we did not observe distinct sex-biased bacterial compositions for samples at the other three age stages in PCoA analysis (Additional file 3: Figure S2). This should be due to the reasons of sexual immuration for piglets at the age of 25 days and the castration for boars at 120 and 240 days. However, this discrepancy may also be caused by the different amplicons in 16S rRNA gene sequencing analysis for samples at 80 days.

### Longitudinal dynamics of enterotype-like clustering of porcine fecal microbiota

**Fig. 3:**
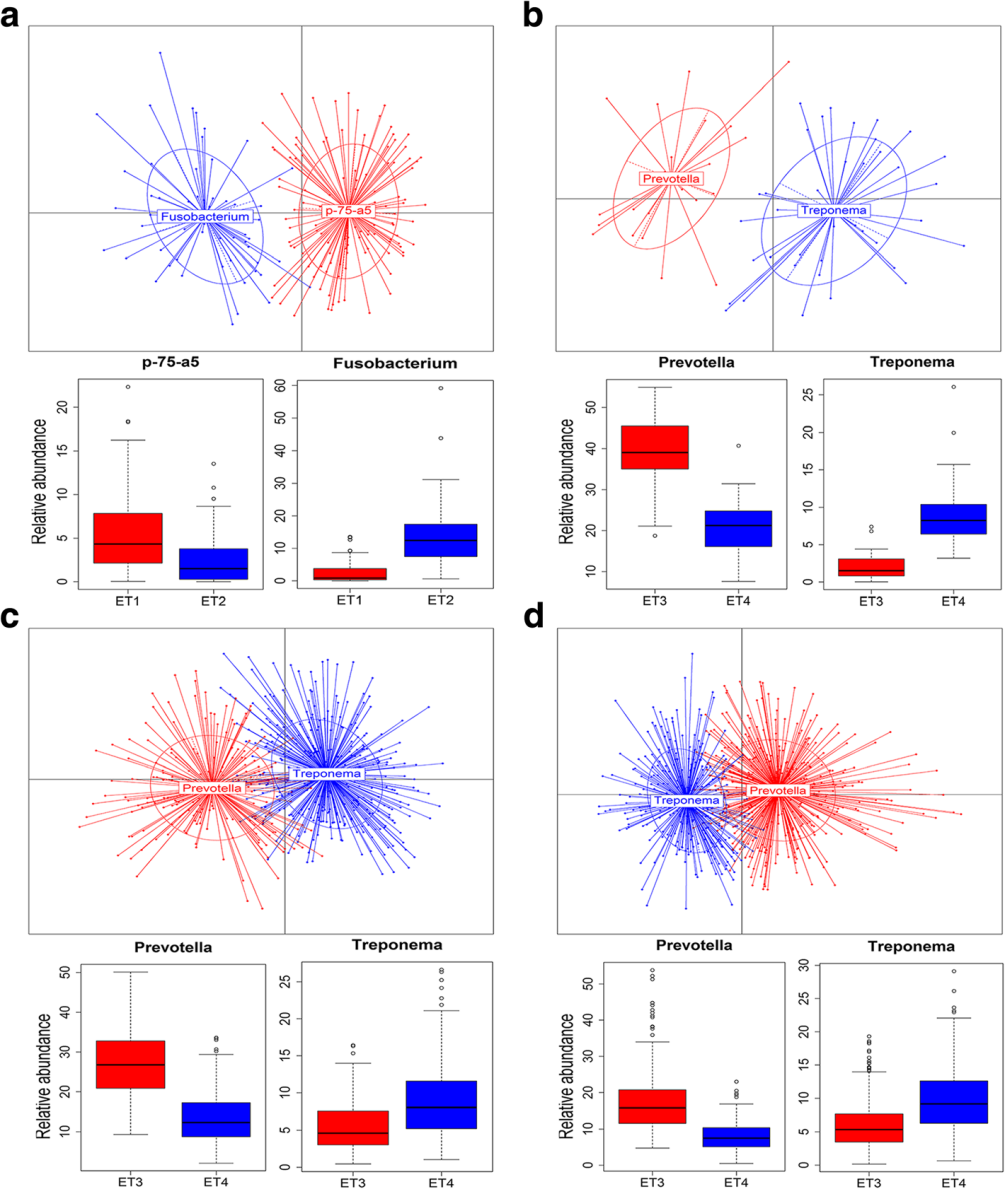
Identification of enterotypes in a cohort of 935 experimental pigs at four ages. (**a**) 25 days. (**b**) 80 days. (**c**) 120 days. (**d**) 240 days. The graphs for enterotype clusters indicate the first two principal coordinates of the Jensen-Shannon distances at different age groups based on the relative abundance profiles at the genus level. Samples are colored by enterotypes as identified by the partitioning around medoids (PAM) clustering algorithm. The percentage of variations explained by PC1 and PC2 are indicated in the X and Y axis, respectively. Boxes represent the relative abundances of the main drivers in each enterotype

We separately performed the enterotype-like clustering analysis at each of four ages. As the result, two enterotype-like clusters were identified for each age. Distinct from the enterotypes for pre-weaning piglets, which were dominated by *p-75-a5* and *Fusobacterium*, respectively (enterotype 1 and 2), the enterotype clusters for the pigs at 80, 120 and 240 days showed a high degree of similarity and could be grouped into the other two enterotypes that were overrepresented by *Prevotella* and *Treponema*, respectively (enterotype 3 and 4) (Fig. 3). The differential taxa of bacteria between the two enterotypes at each age are shown in Additional file 4: Figure S3. To further investigate the discrepancy of the enterotypes between pre-weaning piglets and the pigs at the other three ages, we constructed the SparCC network at the genus level in all four age groups (Additional file 5: Figure S4). We observed that *Fusobacterium* and *p-75-a5* were not only the main drivers for the enterotypes at 25 days (Fig. 3A), but also the hub nodes in the network (Additional file 5: Figure S4). However, *Treponema* was also overrepresented in the enterotype 1, whereas *Prevotella* was more abundant in the enterotype 2 (Additional file 4: Fig. S3), and these two genera were the other two hubs of the network at 25 days. Furthermore, *Fusobacterium* showed co-abundance with *Prevotella* and co-exclusion with *Treponema*. This result suggested that the gut microbiota in pre-weaning piglets should not change completely with age, but only increased the diversity. Some bacterial taxa gradually dominated the bacterial composition by replacing the initial predominant taxa following the age.

To evaluate the stability of enterotypes following the age, we particularly checked the enterotype distribution in pigs with fecal samples at more than one age. Of the 57 pigs having fecal samples at both 25 and 120 days (Additional file 2: Fig. S1), twenty-three (40.35%) were assigned to the *Fusobacterium* enterotype, and the others (59.65%) were occupied by *p-75-a5* enterotype at 25 days of age. However, at the age of 120 days, among those pigs with *Fusobacterium* enterotype at 25 days of age, sixteen (69.57%) switched their putative enterotypes to *Prevotella*, and the others (30.43%) changed their enterotypes to *Treponema*. A similar result was also obtained in pre-weaning pigs with *p-75-a5* enterotype. Twenty-six (76.47%) out of these piglets shifted the enterotypes to *Prevotella*, whereas the left 23.53% of piglets switched the enterotypes to *Treponema* at 120 days. There were 377 pigs involved in the enterotype analysis at both 120 and 240 days (Additional file 2: Figure S1). Eighty-four (22.3%) pigs with *Prevotella* enterotype at 120 days switched their putative enterotypes to *Treponema* at 240 days. Inversely, 142 (37.7%) pigs having *Treponema* enterotype changed the enterotypes to *Prevotella* at 240 days.

### Dynamic changes of interaction networks of porcine fecal bacteria with age

**Fig. 4:**
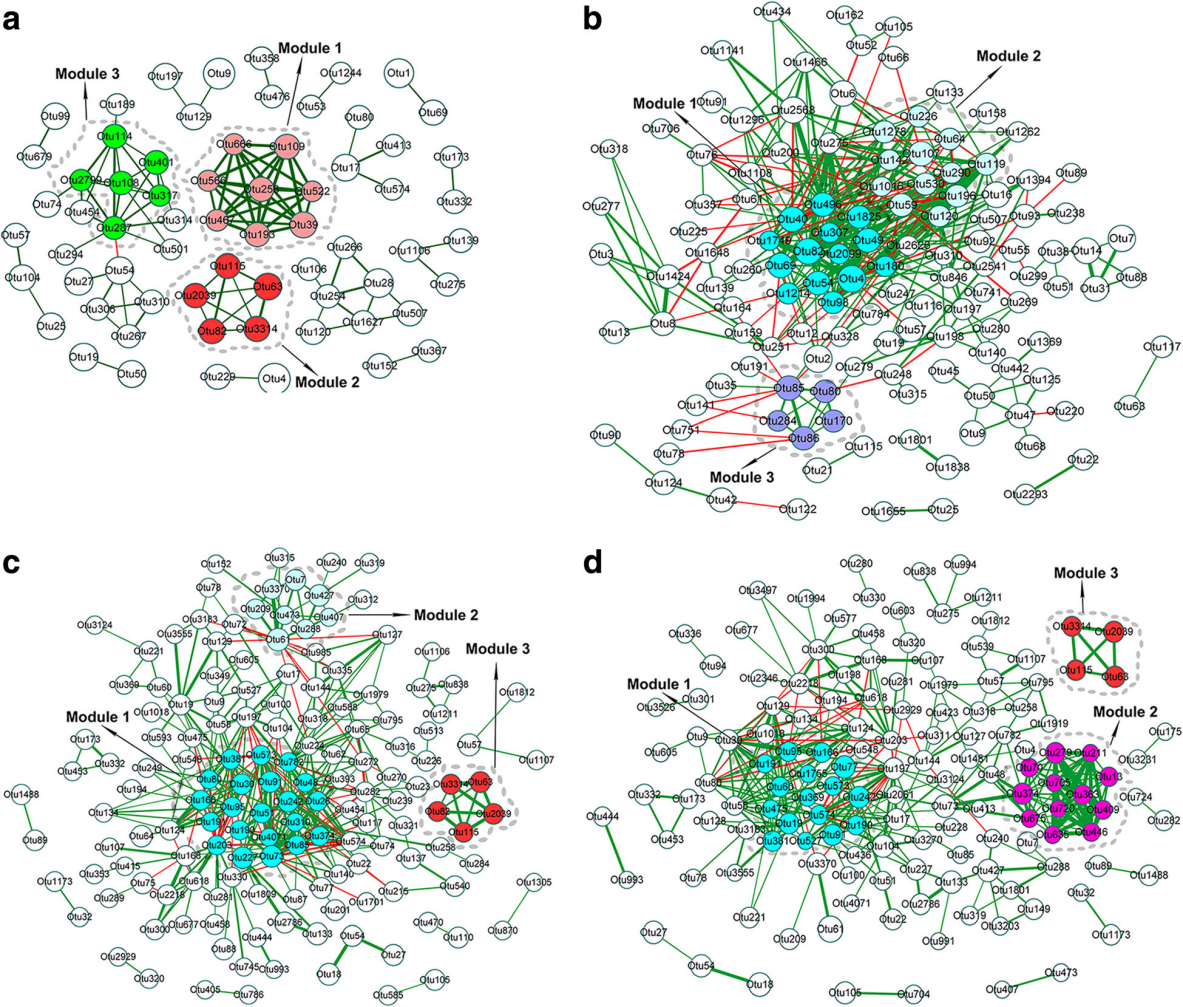
The phylogenetic co-occurrence networks of porcine fecal microbiota at four ages. The networks were constructed at the OTU level. (**a**) 25 days. (**b**) 80 days. (**c**) 120 days. (**d**) 240 days. The size of the nodes shows the abundance of OTUs, and the different colors indicate the modules within networks. The same color shows the similar modules. Edge color represents positive (green) and negative (red) correlations. The edge thickness indicates the correlation values, only the high-confidence interactions with absolute sparse correlations more than 0.55 were selected

**Fig. 5:**
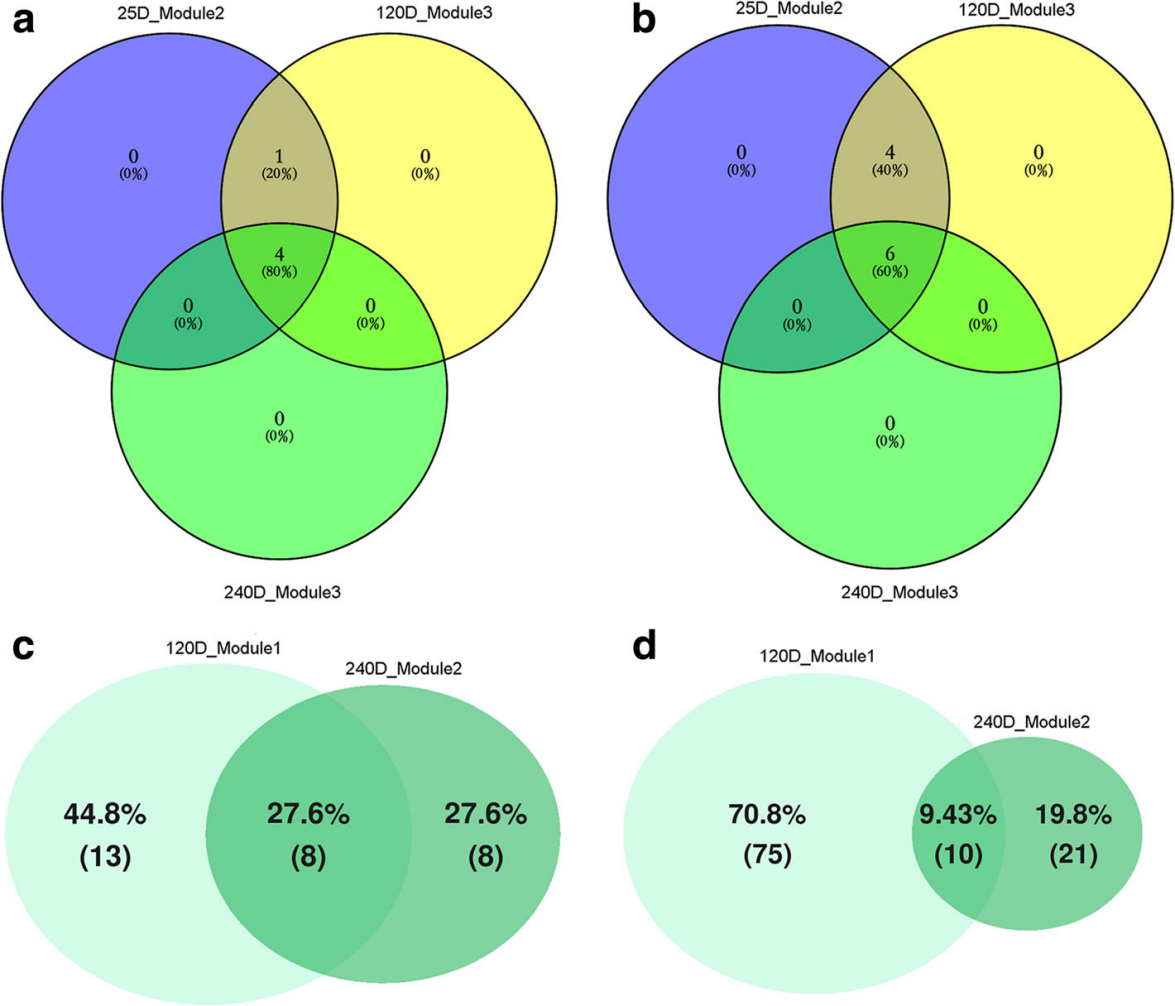
The venn diagram showing the similarity of the main modules in the networks of porcine gut microbiota at different ages based on the number of overlapping nodes and edges. (**a**) The numbers of overlapping nodes among the module 2 at 25 days, module 3 at 120 days and 240 days. (**b**) Overlapping edges among the module 2 at 25 days, module 3 at 120 days and 240 days. (**c**) Overlapping nodes between the module 1 at 120 days and module 2 at 240 days. (**d**) Overlapping edges between the module 1 at 120 days and module 2 at 240 days

To evaluate the dynamic changes of potential interaction networks among bacterial taxa at different ages, 273, 334, 351 and 322 key OTUs at the age of 25, 80, 120 and 240 days were used for constructing the phylogenetic interaction networks, respectively. Overall, in agreement with the previous report [37], OTUs exhibited a various degree of connectivity at each age. The complexity of the phylogenetic interaction networks reflecting in the average number of edges per node was increased from 25 (1.48) to 120 days (2.4), but no significant difference between 120 and 240 days (2.29). The stability (i.e. percentage of negative interactions) was obviously increased from 1.0% at 25 days to 16.9% at 120 days. However, this percentage was decreased to 8.9% at 240 days. The highest percentage of negative interactions was identified at 80 days (21.4%). This continuously declined percentage from the ages of 80 and 120 days to 240 days suggested that the highest stability of gut microbial ecology was achieved before 120 days. However, we could not exclude the influence of the smaller sample size and the different amplicon in 16S rRNA gene sequencing on the percentage of negative interactions for samples at 80 days. In more details, the interaction network of the gut microbiota at the age of 25 days was comprised of 66 nodes and 98 edges (Fig. 4A). This phylogenetic interaction network was mostly divided into three distinct modules, including two piglet-specific modules (module 1 and 3). The nodes in the module 1 were mainly annotated to *Leptonema*, *PSB-M-3*, *Shewanella*, *Pseudomonas* and *Sulfurospirillum* (Additional file 6: Table S2). These bacterial genera are mainly involved in the utilization of carbohydrate in diet milk [38–41]. The module 2 was comprised of *Sphaerochaeta* (Additional file 6: Table S2) which can produce a diverse set of saccharolytic enzymes that participate in the glycolytic and pentose phosphate pathways [42]. Based on the overlapping structures and topological features (Fig. 5 and Additional file 7: Figure S5), this module was also presented at 120 (module 3) and 240 (module 3) days of age. Module 3 contained the bacteria that mainly takes part in the utilization of the lactose and glutamic acid (Additional file 6: Table S2) [43–46]. The interaction network at the age of 80 days was comprised of 129 nodes and 350 interactions. Three modules were identified in this network (Fig. 4B). Most of the nodes in this co-occurrence network belonged to the genera *Blautia*, *Faecalibacterium*, *Prevotella*, *Ruminococcus* and *Treponema* (Additional file 6: Table S2). These microbes are mainly involved in the digestion of dietary polysaccharides (e.g. fiber, cellulose and lignin) and the production of significant amounts of short chain fatty acids (SCFAs) [5, 47–49].

The interaction network at the age of 120 days was comprised of 150 nodes and 360 edges, and mainly divided into three modules (Fig. 4C). Based on the topological features, the module 1 and 3 were highly similar to the module 2 at the age of 240 days and the module 2 at the age of 25 days, respectively (Fig. 5 and Additional file 7: Figure S5). The module 2 consisted of *Prevotella* and *Bacteroides* (Additional file 6: Table S2), which play essential roles in the process of the degradation of complex dietary polysaccharides and amino acid metabolism [47, 50, 51]. The interaction network at the age of 240 days was comprised of 142 nodes and 325 edges. Three distinct modules were identified in this co-occurrence network (Fig. 4D), and only the module 1 which was primarily comprised of the bacteria related to dietary protein metabolism (e.g. *Cetobacterium somerae*, *Coprococcus eutactus*, *Mycobacterium*, *CF231*, *Comamonas*, *Ralstonia*, *Sporosarcina* and *Nitrincola*) was unique to this network [52–59]. The modules 2 and 3 were highly similar to the modules identified at 120 (module 1) and 25 days (module 2), respectively.

### Longitudinal dynamics of predicted function capacity of fecal microbiome

**Fig. 6:**
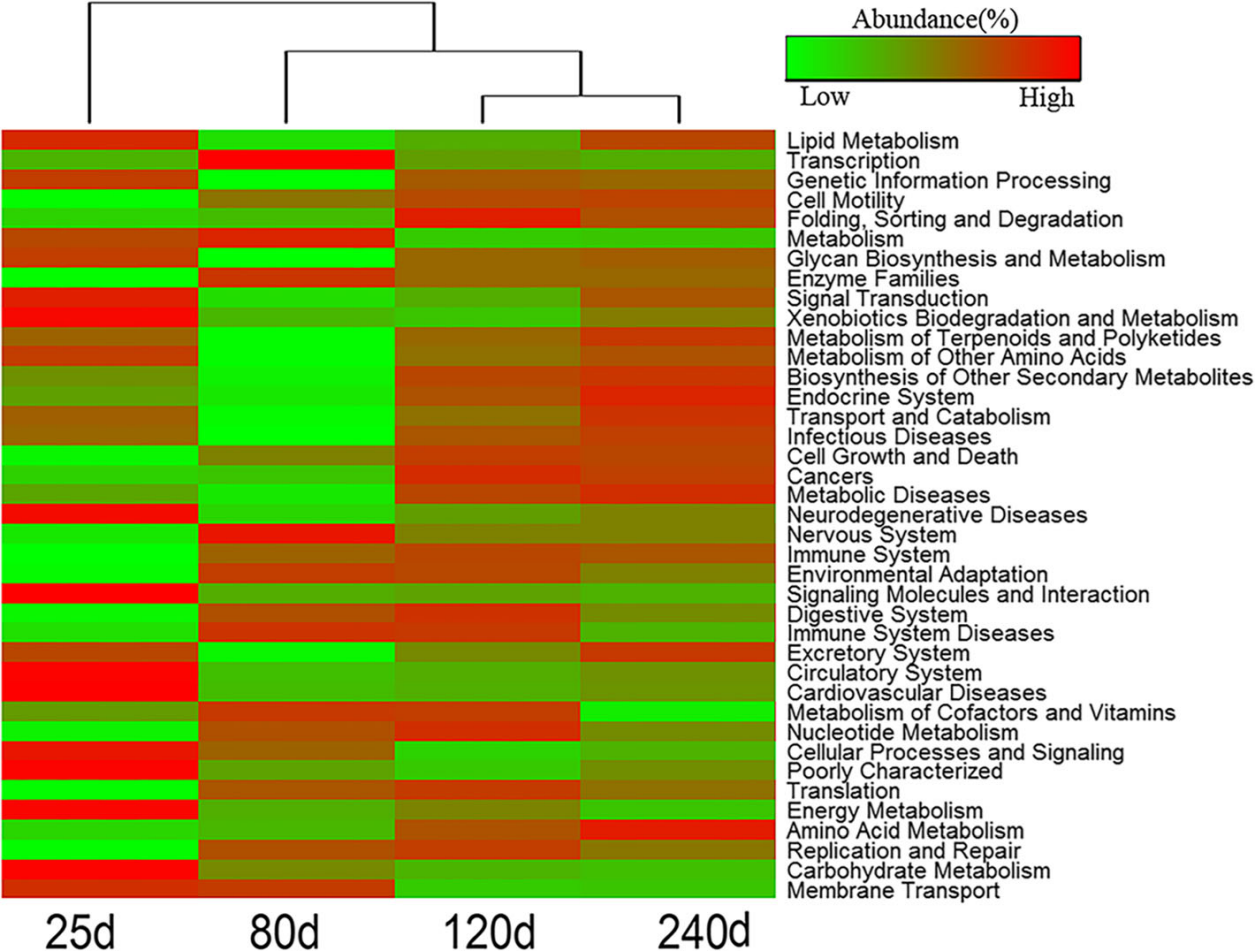
Heatmap for comparing the functional capacities of fecal microbiome among four ages. The color of the bars in the panel represents the relative abundances of the KEGG pathway in each age

To investigate the dynamic changes of the potential function capacity of fecal microbiome with age, the relative abundances of KEGG pathways were predicted by PICRUST based on 16S rRNA gene sequences. A total of 39 KEGG pathways showed significantly different enrichments at different ages (*P* < 0.05, Fig. 6). Eleven out of these 39 KEGG pathways were significantly enriched in pre-weaning piglets (Additional file 8: Table S3), including carbohydrate metabolism, energy metabolism, and xenobiotics biodegradation and metabolism. There were four KEGG pathways showing significant enrichments at the age of 80 days, including enzyme families, metabolism, nervous system and transcription (*P* < 0.001). We also detected one (folding, sorting and degradation, *P* < 0.01) and four (amino acid metabolism, endocrine system, excretory system, and transport and catabolism, *P* < 0.001) pathways that were significantly enriched at 120 and 240 days, respectively. Furthermore, the relative abundances of cell motility and amino acid metabolism were significantly increased following the age (*P* < 0.001), whereas the relative abundance of membrane transport was decreased with age (*P* < 0.001).

## Discussion

Several studies have reported the age-related gut microbial shifts in pigs [16, 60–62]. However, to our knowledge, there are few studies about the dynamic shifts of enterotype-like clusters and interaction networks of gut microbiota following the age in an experimental pig cohort with large sample size. In this study, we explored the longitudinal changes of gut microbial composition, enterotypes, co-occurrence interaction network and potential function capacity following the age in an experimental cohort comprised of 953 pigs. Especially, most of the experimental pigs were continuously collected fecal samples at more than one age. Our study provided an integrated overview of longitudinal changes of the porcine gut microbiome.

In accordance with the previous studies [63, 64], the dominant bacterial phyla in porcine fecal microbiota throughout the experimental period were Firmicutes and Bacteroidetes. The age-based dynamic changes of the relative abundance of Bacteroidetes were mainly contributed by *Bacteroides* and *Prevotella*. The decreased percentage of Fusobacteria was mainly caused by reduced abundance of *Fusobacterium*, while the variation of Spirochaetes was a result of increased abundance of *Treponema*. In agreement with the previous reports [16, 62, 65, 66], Proteobacteria was relatively more abundant in the gut of piglets, but its relative abundance was considerably decreased in the adulthood. The relative abundances of opportunistic pathogens (e.g. *Actinobacillus* and *Helicobacter*) might be reduced following the maturation of pig gut microbiota. It has also been demonstrated that a high diversity of gut microbiota is generally considered related to the host health and fitness, and it is also regard as the sign of the maturation of gut microbiota [67, 68]. We found that the richness of fecal microbiota was continuously increased following the age in this study.

Consistent with the findings from previous report [61], two enterotype-like clusters were identified at each age. *Fusobacterium* and *p-75-a5* were the main drivers of the enterotypes in pre-weaning piglet. The genus *p-75-a5* was a member within the family Erysipelotrichaceae, which was highly correlated with the digestion of protein and fat [69]. As we have well known, milk contained high concentration of protein and fat is the main diet for piglets. It has been reported that *Fusobacterium* was associated with some diseases in animals [70], indicating that opportunistic pathogenic bacteria were commonly presented in suckling piglets. Our data coincides with the fact that the piglet has an immature immune system and thus is unable to manage and control the parasitic invaders [71]. March et al. reported that Ruminococcaceae was the main driver for the only enterotype-like cluster in the 14-day-old suckling pigs [61]. This discrepancy may be caused by the different environments, diets and pig breed [72, 73]. *Prevotella* and *Treponema* were the main drivers of the enterotypes at 80, 120 and 240 days. The previous report also found that *Prevotella* and *Treponema* were the main drivers for the enterotypes of pigs at 60 days of age [37]. *Prevotella* is capable of metabolizing dietary polysaccharide and producing large amounts of SCFAs [74]. *Treponema* plays an essential role in cellulose and lignin degradation [48]. This result suggested that the gut microbiota was mature when the age of pigs was above 80 days, and its ability digesting diet polysaccharide was improved. The enterotypes in pre-weaning pigs did not remain stable (see Results). Surprisingly, the enterotypes did not keep stable even in some adult pigs, e.g. more than 22.3% of pigs with *Prevotella* enterotype at 120 days showed *Treponema* enterotype at 240 days. It has been reported that *Prevotella* enterotype may be better adapted to the diet for growing pigs which contains more plant polysaccharides [37]. In this study, the diets provided to the pigs at 120 and 240 days of age contained slight difference of coarse fiber levels (increased from 5 to 8%) (Additional file 1: Table S1). Costea et al. also reported that although all three enterotypes of human gut microbiota kept stable in overall, 16% of individuals switched putative enterotypes between visits [75].

The scale, complexity and stability of the phylogenetic co-occurrence network at 25 days of age were lower than that at the other three ages, but the networks at 120 and 240 days were highly similar. This was consistent with the result of enterotype analysis, which suggested an unstable structure of fecal microbial community in pre-weaning piglets. The gut microbial ecology would remain stable once the gut microbiota achieves maturation although it may be fluctuations in populations [4]. The main modules in the co-occurrence network of piglets were associated with the utilization of simple carbohydrate (module 1) and lactose (module 3). It is well known that sow milk was the main source of nutrients for suckling piglets. The module commonly identified at 25 (module 2), 120 (module 3) and 240 (module 3) days of age was comprised of *Sphaerochaeta* which has been reported to take part in glucose metabolism through glycolytic and pentose phosphate pathways [42, 76]. This result suggested that glycolytic and pentose phosphate pathways of gut microbiome were essential for pigs throughout the whole-life span. However, this module was absent in the network at 80 days. This discrepancy may be caused by the smaller sample size and the different sequencing region of 16S rRNA gene. Several modules identified at 80, 120 (module 1 and 2) and 240 (module 2) days were related to the digestion of nondigestible carbohydrates and the production of SCFAs [5, 49], suggesting that the gut microbiota above the age of 80 days obtained the functional capacity of digesting dietary fiber. The modules involved in the metabolism of dietary protein and amino acids were identified at 120 (module 2) and 240 (module 1) days, indicating that the functional capacity of gut microbiome digesting dietary protein had been significantly improved since the age of 120 days.

Consistent with the result observed in the interaction network analysis, the KEGG pathways enriched in piglets were related to carbohydrate and energy metabolism. This may be due to the high fat and carbohydrate concentration of sow milk diet. The functional capacities of cell motility and amino acid metabolism were increased with ages. Cellular motility is essential for biological processes, such as the development and immune response [77]. As mentioned above, the abilities of immune response and amino acid metabolism of gut microbiome were increased following the age.

## Conclusions

The present study demonstrated the dynamic changes of the phylogenetic composition, enterotypes, interaction networks and potential functional capacity of porcine gut microbiome following the age. These longitudinal changes of gut microbiome contribute to the metabolism of diet nutrients and the maturation of immune system in pigs. The results provide meaningful biological insights into the age-based dynamic shifts of ecological community of porcine gut microbiota and provide valuable knowledge about establishment and maturation of gut microbiome.

## Additional files

Additional file 1: **Table S1.** Summary description of dietary components for pigs at different ages. (XLSX 9 kb)

Additional file 2: **Figure S1.** The venn diagram showing the sample distribution of experimental pig cohort among four ages. (TIF 269 kb)

Additional file 3: **Figure S2.** PCoA showing the effect of sex on fecal bacterial composition based on the Bray-Curtis similarity matrix. (TIF 3183 kb)

Additional file 4: **Figure S3.** Histogram of the linear discriminant analysis (LDA) score for differentially abundant genera between the enterotypes. (A) 25 days. (B) 80 days. (C) 120 days. (D) 240 days. Genera with LDA scores > 3 were presented. (TIF 823 kb)

Additional file 5: **Figure S4.** The SparCC network at the genus level showing that the main driver of each enterotype was also the hub node. (A) 25 days. (B) 80 days. (C) 120 days. (D) 240 days. The size of the nodes represents the relative abundance of each genus. Node colors indicate the phylum that each genus belongs to. Edge colors represent positive (green) and negative (red) correlations, and the thickness of the edges indicates the values of the correlations. (TIF 1973 kb)

Additional file 6: **Table S2.** Summary of annotation information of node OTUs from biologically important modules in the phylogenetic co-occurrence networks at four ages. (XLSX 15 kb)

Additional file 7: **Figure S5.** Comparison of the topological features for main modules in the networks of porcine gut microbiota at different ages. (TIF 146 kb)

Additional file 8: **Table S3.** Comparison of predicted functional capacities of fecal microbiome among four ages. (XLSX 15 kb)

## Abbreviations

KEGG: Kyoto encyclopedia of genes and genomes
OTU: Operational taxonomic units
rRNA: Ribosomal robonucleic acid
SCFA: Short-chain fatty acid
