# Supplementary figures and images for: Age-based dynamic changes of phylogenetic composition and interaction networks of health pig gut microbiome feeding in a uniformed condition
Source: BMC Vet Res. 2019 May 24;15:172. doi: 10.1186/s12917-019-1918-5 (PMC6534858; doi:10.1186/s12917-019-1918-5)

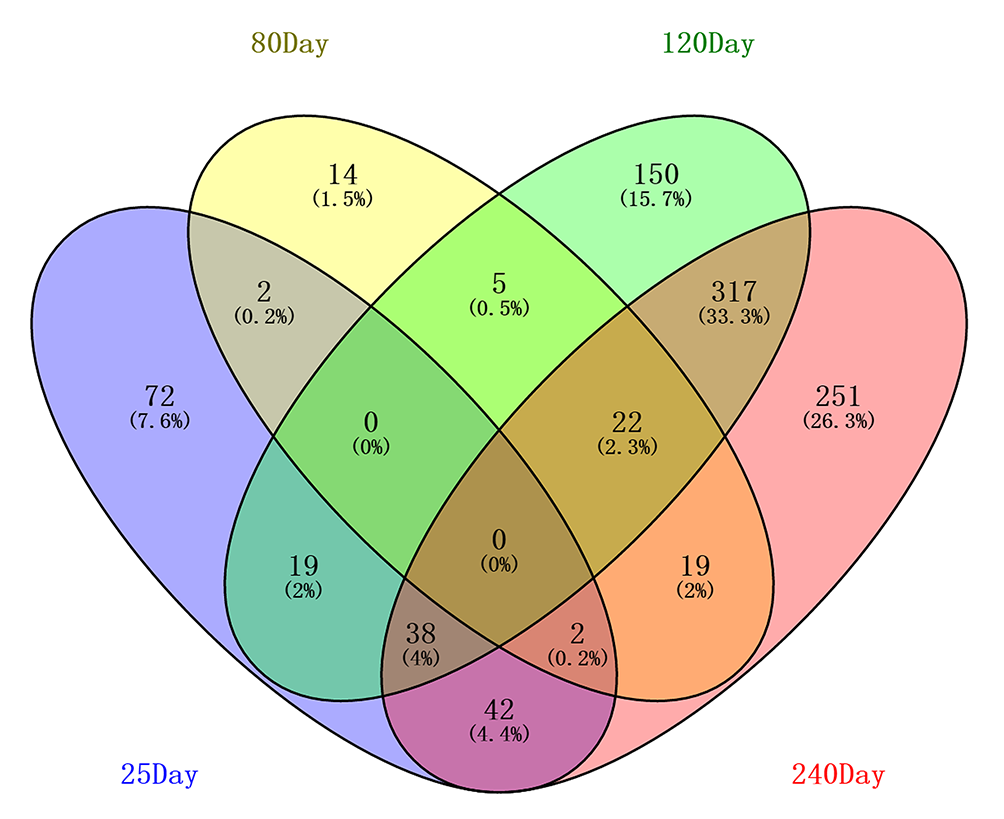

Supplement: Supplementary file 2 — Figure S1. The venn diagram showing the sample distribution of experimental pig cohort among four ages. (TIF 269 kb) [file 12917_2019_1918_MOESM2_ESM.tif]

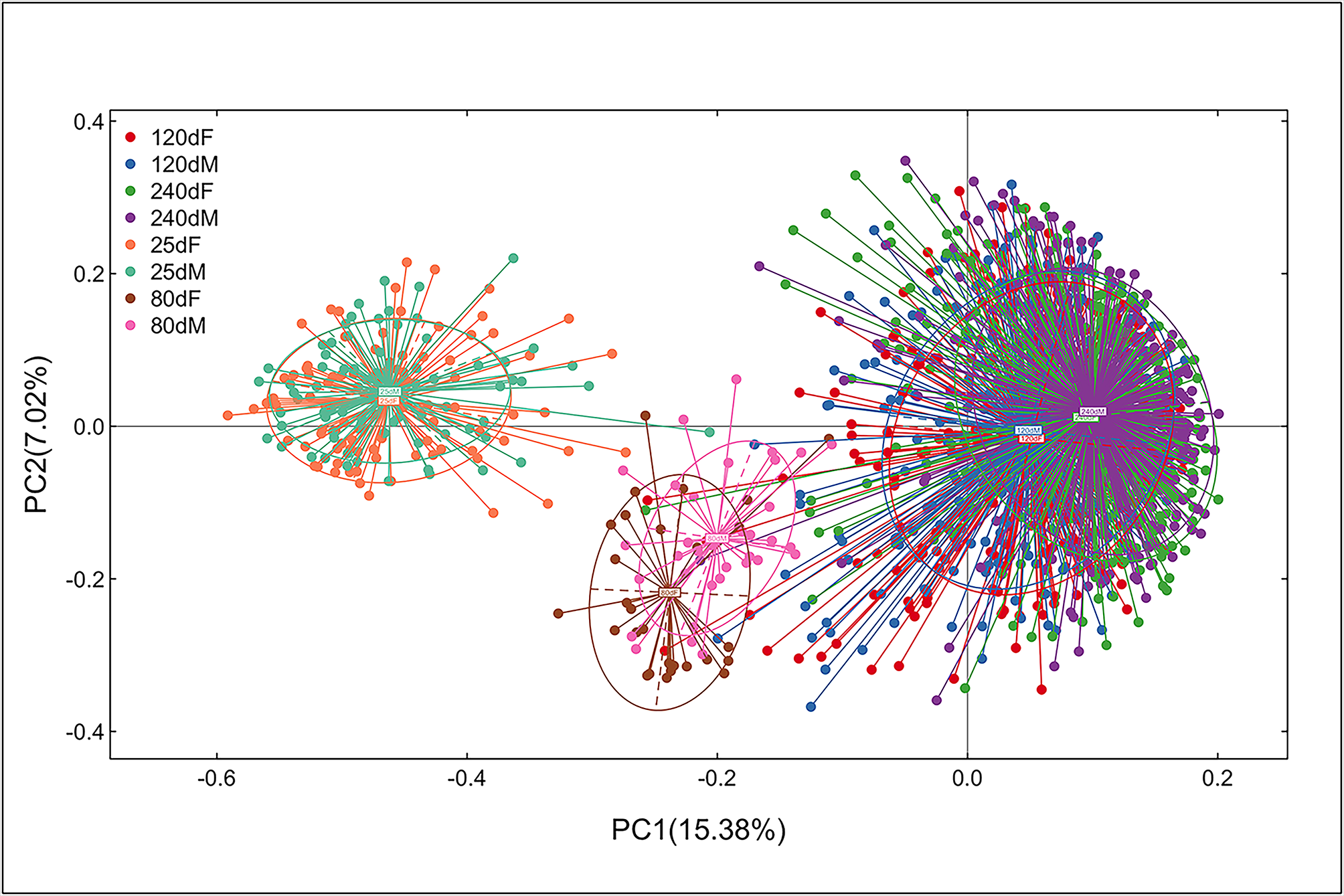

Supplement: Supplementary file 3 — Figure S2. PCoA showing the effect of sex on fecal bacterial composition based on the Bray-Curtis similarity matrix. (TIF 3183 kb) [file 12917_2019_1918_MOESM3_ESM.tif]

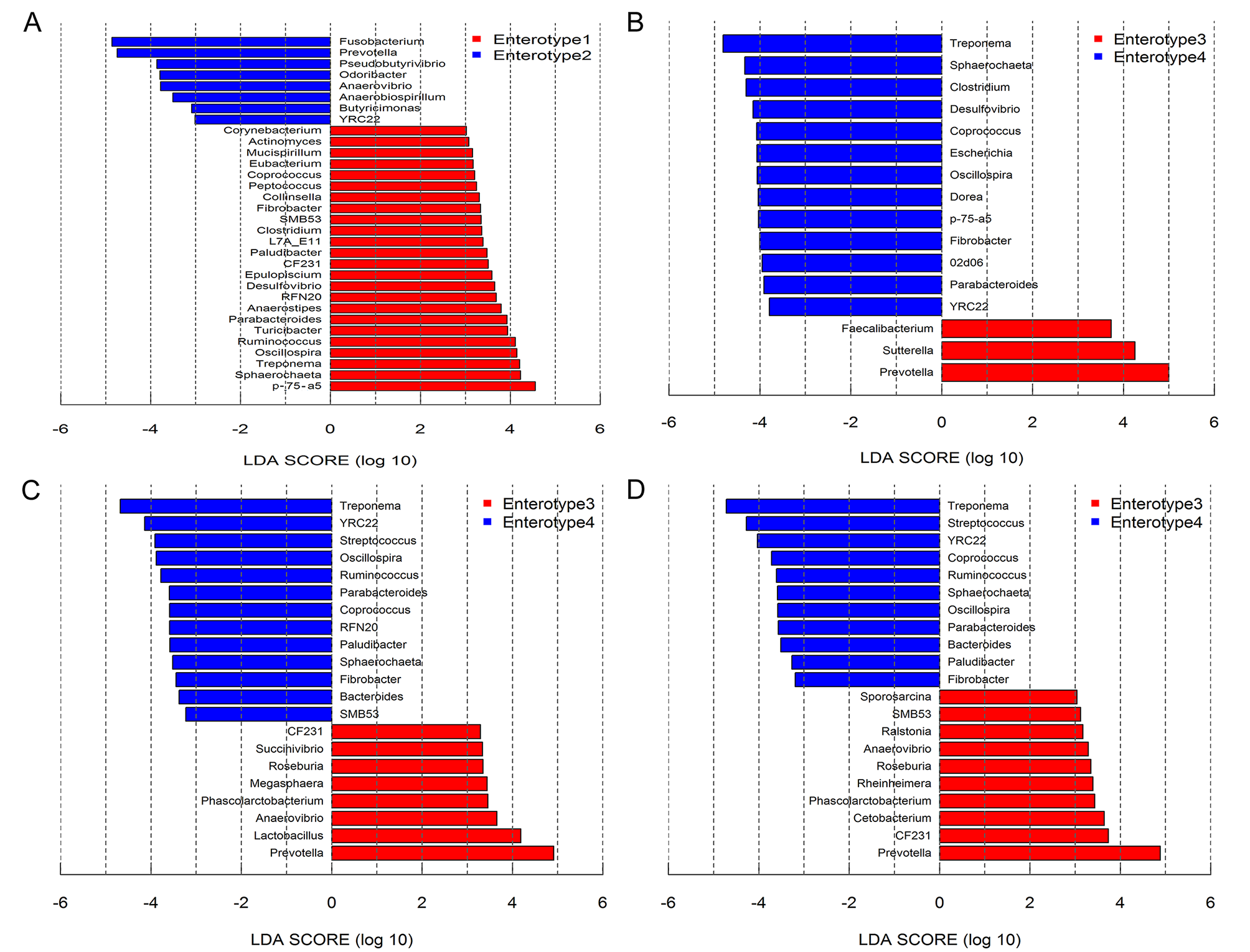

Supplement: Supplementary file 4 — Figure S3. Histogram of the linear discriminant analysis (LDA) score for differentially abundant genera between the enterotypes. (A) 25 days. (B) 80 days. (C) 120 days. (D) 240 days. Genera with LDA scores > 3 were presented. (TIF 823 kb) [file 12917_2019_1918_MOESM4_ESM.tif]

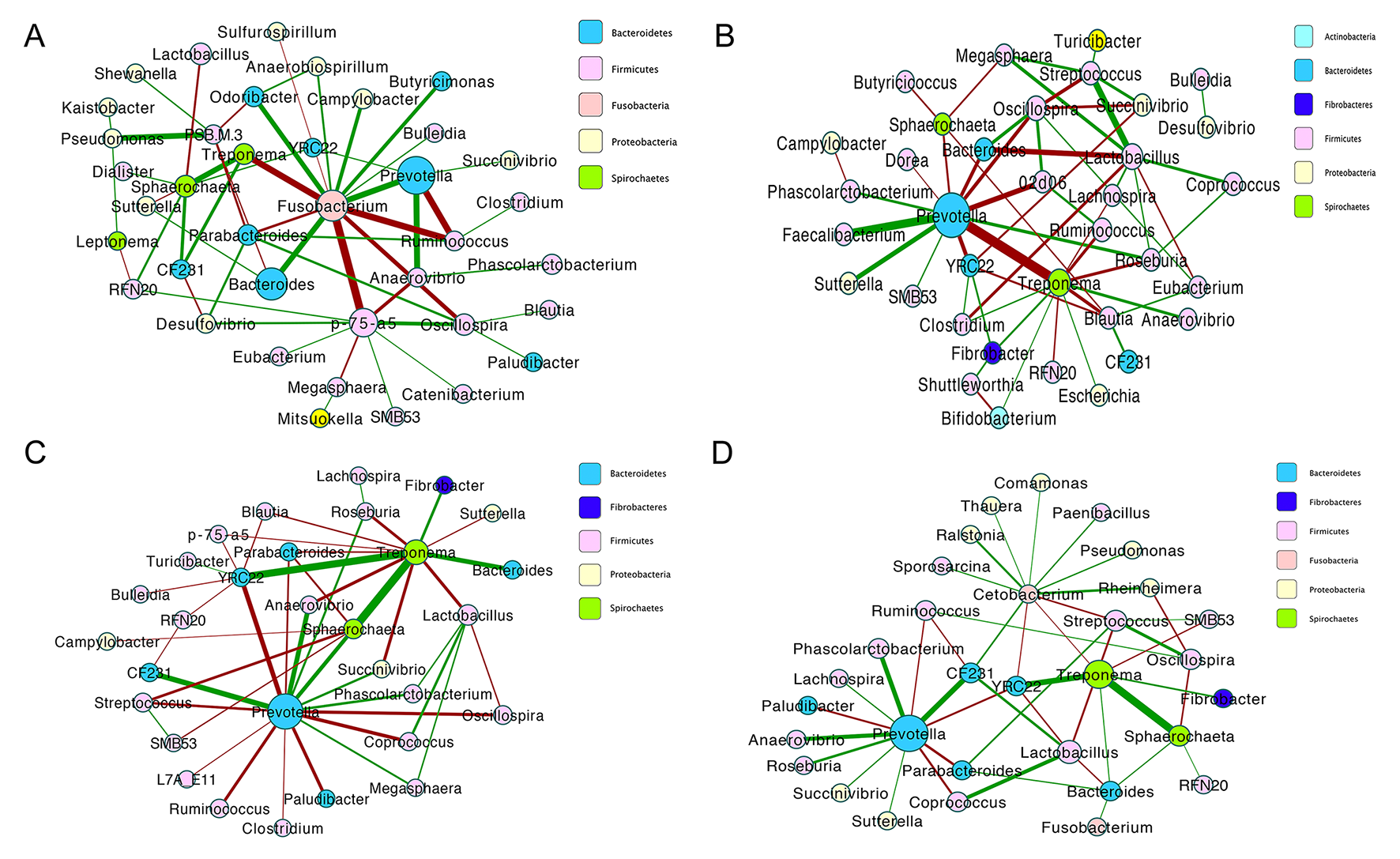

Supplement: Supplementary file 5 — Figure S4. The SparCC network at the genus level showing that the main driver of each enterotype was also the hub node. (A) 25 days. (B) 80 days. (C) 120 days. (D) 240 days. The size of the nodes represents the relative abundance of each genus. Node colors indicate the phylum that each genus belongs to. Edge colors represent positive (green) and negative (red) correlations, and the thickness of the edges indicates the values of the correlations. (TIF 1973 kb) [file 12917_2019_1918_MOESM5_ESM.tif]

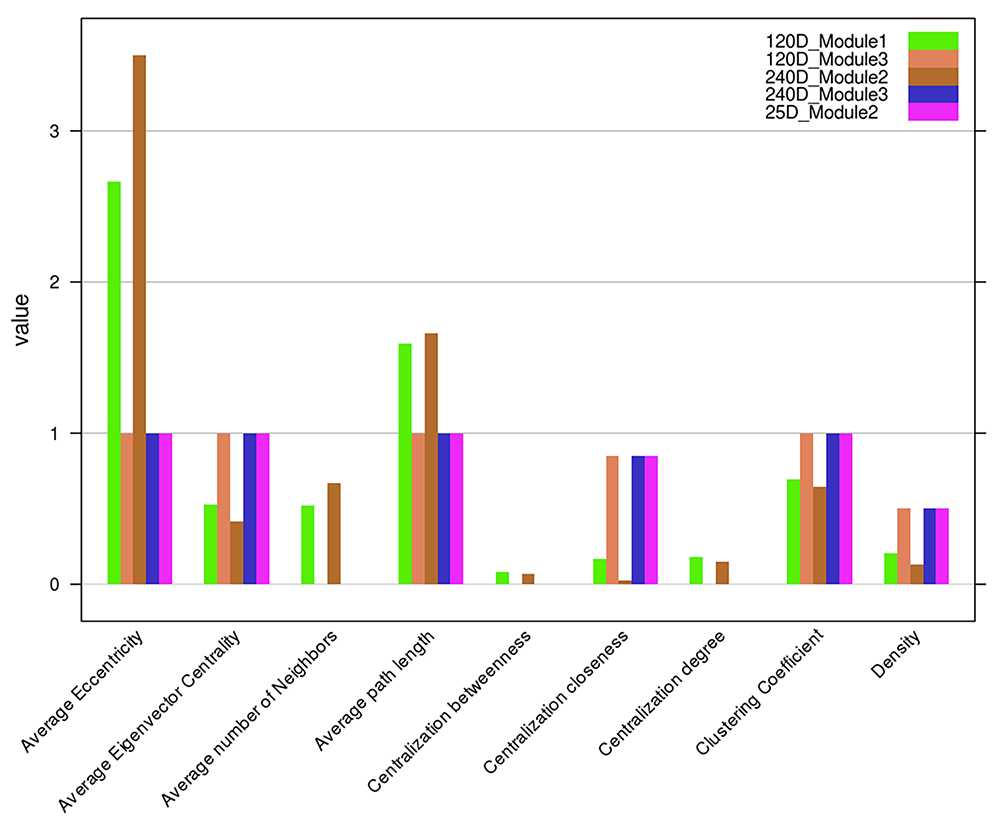

Supplement: Supplementary file 7 — Figure S5. Comparison of the topological features for main modules in the networks of porcine gut microbiota at different ages. (TIF 146 kb) [file 12917_2019_1918_MOESM7_ESM.tif]
